# Rates and risk factors associated with hospitalization for pneumonia with ICU admission among adults

**DOI:** 10.1186/s12890-017-0552-x

**Published:** 2017-12-16

**Authors:** Aaron D. Storms, Jufu Chen, Lisa A. Jackson, James D. Nordin, Allison L. Naleway, Jason M. Glanz, Steven J. Jacobsen, Eric S. Weintraub, Nicola P. Klein, Paul M. Gargiullo, Alicia M. Fry

**Affiliations:** 1 Influenza Division, Centers for Disease Control and Prevention, 1600 Clifton Rd NE, Mailstop A32, Atlanta, GA 30333 USA; 2 Epidemic Intelligence Service, Centers for Disease Control and Prevention, Atlanta, USA; 3 Group Health Cooperative, Seattle, USA; 4 Health Partners Institute, Minneapolis, USA; 5 Center for Health Research, Kaiser Permanente Northwest, Portland, USA; 6 Kaiser Permanente of Colorado, Denver, USA; 7 Kaiser Permanente of Southern California, Pasadena, USA; 8 Immunization Safety Office, Centers for Disease Control and Prevention, Atlanta, USA; 9 Kaiser Permanente of Northern California, Oakland, USA; 10 Present Address: Keck School of Medicine of USC, 2020 Zonal Avenue, IRD 306, Los Angeles, CA 90033 USA

**Keywords:** Pneumonia/epidemiology, Intensive care units, Hospitalization, Trends, Rates, Adult

## Abstract

**Background:**

Pneumonia poses a significant burden to the U.S. health-care system. However, there are few data focusing on severe pneumonia, particularly cases of pneumonia associated with specialized care in intensive care units (ICU).

**Methods:**

We used administrative and electronic medical record data from six integrated health care systems to estimate rates of pneumonia hospitalizations with ICU admissions among adults during 2006 through 2010. Pneumonia hospitalization was defined as either a primary discharge diagnosis of pneumonia or a primary discharge diagnosis of sepsis or respiratory failure with a secondary diagnosis of pneumonia in administrative data. ICU admissions were collected from internal electronic medical records from each system. Comorbidities were identified by ICD-9-CM codes coded during the current pneumonia hospitalization, as well as during medical visits that occurred during the year prior to the date of admission.

**Results:**

We identified 119,537 adult hospitalizations meeting our definition for pneumonia. Approximately 19% of adult pneumonia hospitalizations had an ICU admission. The rate of pneumonia hospitalizations requiring ICU admission during the study period was 76 per 100,000 population/year; rates increased for each age-group with the highest rates among adults aged ≥85 years. Having a co-morbidity approximately doubled the risk of ICU admission in all age-groups.

**Conclusions:**

Our study indicates a significant burden of pneumonia hospitalizations with an ICU admission among adults in our cohort during 2006 through 2010, especially older age-groups and persons with underlying medical conditions. These findings reinforce current strategies aimed to prevent pneumonia among adults.

**Electronic supplementary material:**

The online version of this article (10.1186/s12890-017-0552-x) contains supplementary material, which is available to authorized users.

## Background

Pneumonia poses a significant burden to the U.S. health-care system. In 2010, pneumonia was one of the 10 leading causes of death in the U.S. [1] and resulted in over 50,000 deaths (a rate of 16.2 per 100,000 in the U.S. population) [2]. Approximately 1.1 million pneumonia associated hospital discharges (a rate of 366 per 100,000 U.S. population) have been reported [3]. However, there are few data focusing on severe pneumonia, such as cases of pneumonia requiring care in an intensive care unit (ICU), though these data could facilitate planning for resource utilization (i.e. use of hospital beds, ICU beds, and ventilators) during emergencies. We used administrative and electronic medical record data from six integrated health care systems to estimate rates of pneumonia hospitalizations with ICU admissions among adults during 2006 through 2010.

## Methods

The Vaccine Safety Datalink (VSD) is a joint project between the Centers for Disease Control and Prevention (CDC) and eight integrated health care systems in the U.S. We used data from six of the eight VSD sites for this study (Group Health Cooperative [Washington], HealthPartners Institute [Minnesota], Kaiser Permanente of Colorado, Kaiser Permanente of Northern California, Kaiser Permanente Northwest [Colorado, California, Oregon], and Kaiser Permanente of Southern California), which comprises approximately 6 million enrollees per year (2.5% of the 2010 U.S. population). Two sites were excluded as they did not consistently capture ICU admissions. Data collected from patients aged ≥18 years enrolled in these sites include demographics (age, sex), dates of out-patient medical encounters or hospital admissions, dates of discharge, and International Classification of Diseases, Ninth Revision, Clinical Modification (ICD-9-CM) diagnosis and procedure codes for all out-patient and in-patient medical encounters [4].

Pneumonia hospitalization was defined by an expanded case definition [5] which included any of the following three possibilities: 1) primary discharge diagnosis of pneumonia (ICD-9-CM codes: 480–488); 2) primary discharge diagnosis of sepsis (ICD-9-CM codes: 038, 003.1, 020.2, 022.3, 036.2, 036.3, 054.5, 098.89, 785.52, 995.91, 995.92) with pneumonia listed as a secondary diagnosis in any diagnostic field; 3) primary discharge diagnosis for respiratory failure (ICD-9-CM codes: 518.81, 518.82, 518.84, 799.1) with pneumonia listed as a secondary diagnosis in any diagnostic field. Note that we added the influenza ICD-9-CM codes (487 and 488), which included seasonal and novel influenza viruses (i.e., H1N1pdm09), influenza pneumonia, as well as other influenza associated syndromes to the previously published “expanded pneumonia” codes as influenza can be associated with lower respiratory tract illness. Very few hospitalizations had influenza discharge codes (data not shown). We included persons aged ≥18 years hospitalized during January 1, 2006 through December 31, 2010.

ICU admission was defined as an admission to any of the following hospital units during the pneumonia hospitalization: pediatric ICU, medical ICU, cardiac care unit, surgical ICU, neuro/neurosurgical ICU, transition or step-down unit. ICU admissions were identified by each of the sites using their internal electronic medical records and site-specific methods to query the electronic medical record for ICU admissions. We also assessed “assisted ventilation”, which included invasive assisted ventilation and non-invasive interventions such as BiPAP and CPAP. We used assisted ventilation current procedure terminology (CPT) and ICD-9-CM procedure codes for assisted ventilation use (Additional file 1: Table S1). Monthly rates were calculated by dividing the monthly number of hospitalizations by the number of active enrollees during each month of the study period. Annual rates were calculated by dividing the number of hospitalizations each year by the yearly average of enrollees. 95% confidence intervals (CI) were calculated around the rate estimates by using the Rothman-Greenland method [6]. Annual rates were age-group adjusted using the 2006 VSD population to standardize the proportion of each age-group for other years.

We assessed the association of age-group (18–49, 50–64, 65–74, 75–84, ≥85 years), sex, and comorbidities with ICU admission. Race and ethnicity data were not available. Comorbidities were identified by ICD-9-CM codes coded during medical visits that occurred during the year prior to the date of admission (i.e. in-patient and out-patient visits, including to the emergency department) as well as during the current pneumonia hospitalization. These included respiratory, cardiovascular, renal and liver diseases, diabetes, malignancies, immunosuppressive, hematological and neurological/musculoskeletal disorders (Additional file 2: Table S2). Crude odds-ratios (OR) were calculated for each comorbidity, in addition to male sex. Those factors that had a *P* value ≤0.2 were included in the multivariable logistic regression model. We assessed for effect modification due to age-group, and for interaction between diabetes, cardiovascular disease and renal disease. All analyses were conducted using SAS 9.3 (Cary, NC).

The Institutional Review Boards in each of the participating institutions reviewed and approved this study. No informed consent was required for this project.

## Results

During January 1, 2006 through December 31, 2010, we identified 119,537 adult hospitalizations meeting our expanded case definition for pneumonia (Table 1). Most pneumonia hospitalizations were from the Northern California (36%) and Southern California (42%) regions of Kaiser Permanente while the remaining 22% were from the other four sites (range 4.3% – 7.2%). Of the included hospitalizations, only 87,457 (73%) met the definition on the basis of a primary discharge diagnosis code of pneumonia, with the remainder meeting the definition on the basis of a primary discharge diagnosis of sepsis or respiratory failure and a secondary diagnosis of pneumonia (Additional file 3: Table S3). The rate of pneumonia hospitalization was 400 per 100,000 population/year for all adults. Among adults hospitalized for pneumonia, the median length of hospital stay was 4 days (interquartile range [IQR] 2–7 days). For patients hospitalized with pneumonia and admitted to the ICU, the median length of hospital stay was 6 days (IQR 4–12 days) with only minor variation by age-group (data not shown).

**Table 1:** Number and rates of pneumonia hospitalizations and pneumonia hospitalizations with an ICU admission—Vaccine Safety Data Link (VSD), 2006–2010

	Pneumonia hospitalizations No. (% of total)	Pneumonia hospitalizations with ICU admissions No. (% of hospitalizations)	Hospitalizations with assisted ventilation No. (% of hospitalizations)	Rates of pneumonia hospitalizations^a^ (95% CI)	Rates of pneumonia hospitalization with ICU admissions^a^ (95% CI)	Rates of pneumonia hospitalizations with assisted ventilation^a^ (95% CI)
Overall	119,537 (100)	22,658 (18.9)	15,564 (13.0)	400.0 (397.7–402.3)	75.8 (74.8–76.8)	52.1 (51.3–52.9)
Age-groups (years)						
18–49	13,999 (11.7)	2750 (18.3)	1772 (12.7)	82.8 (81.5–84.2)	15.2 (14.6–15.8)	10.5 (10.0–11.0)
50–64	24,893 (20.8)	5227 (21.0)	3862 (15.5)	305.3 (301.6–309.2)	64.1 (62.4–65.9)	47.4 (45.9–48.9)
65–74	24,724 (20.7)	5183 (21.0)	3843 (15.5)	901.7 (890.5–913.0)	189.0 (183.9–194.2)	140.2 (135.8–144.7)
75–84	32,880 (27.5)	6010 (18.3)	4006 (12.2)	2105.7 (2083.1–2128.6)	384.9 (375.3–394.7)	256.6 (248.7–264.6)
≥ 85	23,041 (19.3)	3668 (15.9)	2081 (9.0)	4368.4 (4312.3–4425.2)	695.4 (673.3–718.3)	394.5 (377.9–411.9)
Sex^b^						
Male	58,983 (49.4)	11,508 (19.5)	8018 (13.6)	419.4 (416.0–422.8)	81.8 (80.3–83.3)	57.0 (55.8–58.3)
Female	60,548 (50.6)	11,149 (18.4)	7544 (12.5)	382.7 (379.7–385.8)	70.5 (69.2–71.8)	47.7 (46.6–48.8)

^a^Rates per 100,000 population per year

^b^Sex was missing for six persons

ICU = Intensive care unit

Assisted ventilation includes noninvasive and invasive mechanical ventilation

Approximately 19% of adult pneumonia hospitalizations had an ICU admission and 13% had assisted ventilation (Table 1). Not all assisted ventilation was associated with an ICU admission code. Among 15,564 hospitalizations with assisted ventilation, 9557 (61%) also had ICU admission. Overall, 42% (9557/22,658) of pneumonia hospitalizations with ICU admission also had assisted ventilation. The highest proportion of ICU admissions occurred in the patients with a primary respiratory failure discharge code (Additional file 3: Table S3).

The rate of pneumonia hospitalization with ICU admission was 76 per 100,000 population/year for all adults (Table 1). The rates of pneumonia hospitalization, hospitalization with an ICU admission, and hospitalization with assisted ventilation increased for each age group with the lowest rates among adults aged 18–49 years. Rates for adults aged ≥85 years were highest, with 53 times higher rates for pneumonia hospitalizations (4368 per 100,000), and 46 times higher rates for pneumonia hospitalizations with ICU admissions (695 per 100,000), compared to persons in the 18–49 age-group (83 per 100,000 for pneumonia hospitalizations, and 15 per 100,000 for hospitalizations with an ICU admission). Pneumonia hospitalization rates for all persons aged ≥65 years were 1669 per 100,000. The oldest age group (≥85 years) had the lowest proportion of hospitalizations with an ICU admission and assisted ventilation. Rates were higher for males than for females for pneumonia hospitalizations (419 per 100,000 versus 383 per 100,000) and for hospitalizations with ICU admissions (82 per 100,000 versus 71 per 100,000) .

Annual rates of pneumonia hospitalization with ICU admissions ranged between 65.5 and 85.8 per 100,000. Rates during 2010 were higher than 2006. Rates were always higher in older age-groups, regardless of the year (Table 2).

**Table 2:** Rates of pneumonia hospitalizations, pneumonia hospitalizations with an ICU admission, and pneumonia hospitalizations with assisted ventilation by year and age-group —Vaccine Safety Data Link (VSD), 2006–2010

Years and age-groups	Rates of pneumonia hospitalizations^a^ (95% CI)		Rates of pneumonia hospitalization with ICU admissions^a^ (95% CI)		Rates of pneumonia hospitalizations with assisted ventilation^a^ (95% CI)	
2006	376.7	(371.7–381.7)	69.1	(67.0–71.2)	47.9	(46.2–49.8)
18–49 years	67.3	(64.6–70.2)	12.1	(11.0–13.4)	8.6	(7.6–9.6)
50–64 years	279.3	(271.1–289.9)	55.1	(51.5–57.6)	42.5	(39.3–42.7)
65–74 years	886.5	861.4–912.4)	182.5	(171.3–194.4)	136.4	(126.8–146.8)
75–84 years	2122.6	(2071.8–2174.7)	371.5	(350.6–393.7)	252.9	(235.7–271.3)
≥ 85 years	4539.7	(4405.4–4678.1)	721.8	(669.4–778.3)	388.1	(250.2–430.1)
2007	367.7	357.1–375.9)	65.5	(61.2–70.2)	46.2	(42.6–50.2)
18–49 years	70.3	(67.5–73.1)	11.9	(10.8–13.1)	9.2	(7.8–9.7)
50–64 years	280.3	(272.2–278.5)	58.6	(54.9–62.4)	41.9	(43.5–50.1)
65–74 years	880.2	(855.2–905.9)	173.5	(162.6–185.1)	126	(135.4–155.8)
75–84 years	2063.4	(2013.1–2114.9)	355.7	(335.2–377.5)	249.6	(239.4–275.2)
≥ 85 years	4085.7	(3961.9–4213.3)	554.2	(509.8–602.5)	332.9	(364.7–441.6)
2008	394.6	(383.7–403.5)	72.4	967.8–77.3)	50.4	(46.6–54.5)
18–49 years	78.2	(75.2–81.2)	13	(11.9–14.3)	8.7	(7.8–9.8)
50–64 years	298.7	(290.5–298.6)	61.5	(58.7–65.4)	46.7	(43.5–50.1)
65–74 years	937.4	(911.9–963/7)	189.9	(178.6–202.0)	145.2	(135.4–155.8)
75–84 years	2164.6	(2113.3–2217.2)	384.2	(362.9–406.7)	256.7	(239.4–275.2)
≥ 85 years	4521.5	(4394.5–4652.2)	707.1	(657.9–759.9)	401.3	(364.7–441.6)
2009	417.7	(406.5–429.1)	85.8	(80.8–91.0)	57.3	(53.3–61.6)
18–49 years	112	(108.4–15.6)	22.7	(21.2–24.3)	15.3	(14.0–16.7)
50–64 years	358.3	(349.2–367.5)	78.5	(74.3–82.9)	56.3	(52.8–60.0)
65–74 years	928.8	(903.9–954.3)	207.5	(195.9–219.7)	150.2	(140.4–160.7)
75–84 years	2059.2	(2009.7–2109.9)	405.2	(383.5–428.0)	262.3	(245.0–280.8)
≥ 85 years	4153.7	(4035.8–4275.1)	744.6	(695.6–797.0)	390.3	(355.2–428.7)
2010	394.32	(383.7–405.2)	77.2	(72.6–82.1)	52.8	(49.0–56.9)
18–49 years	86.7	(83.6–89.9)	16.3	(15.0–17.8)	10.7	(9.6–11.8)
50–64 years	308	(299.8–316.5)	66.3	(62.5–70.3)	49.1	(45.9–52.6)
65–74 years	876.1	(852.6–900.3)	190.3	(179.5–201.7)	141.9	(132.6–151.8)
75–84 years	2118.9	(2069.3–2169.6)	406.2	(382.8–428.8)	260.9	(243.9–279.1)
≥ 85 years	4537.3	(4417.4–4660.4)	736.6	(689.2–787.2)	449.6	(412.9–589.5)

^a^Rates per 100,000 population per year. Yearly summary rates are age adjusted

ICU = Intensive care unit

**Fig. 1:**
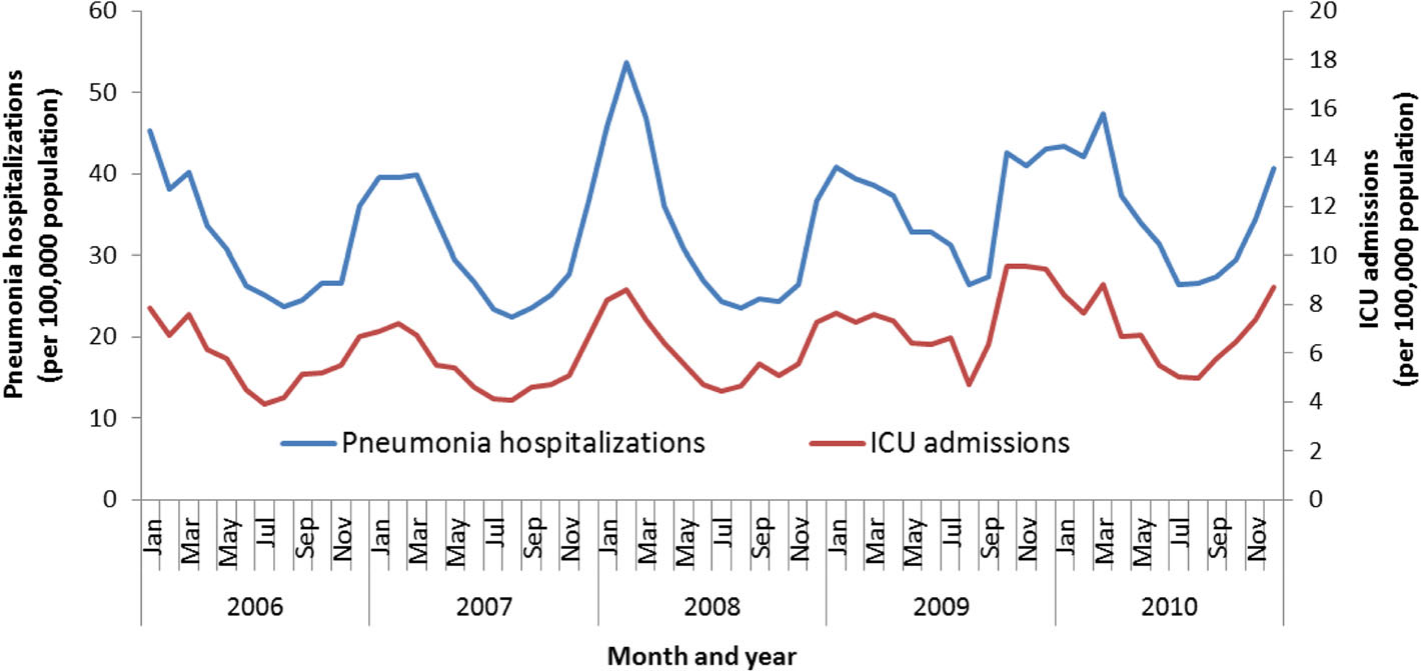
Monthly rates of pneumonia hospitalizations and associated ICU admissions—Vaccine Safety Data Link (VSD), 2006–2010

The monthly rates of pneumonia hospitalization with ICU admission were consistently highest in the winter months during the 5-year study period, similar to pneumonia hospitalizations (Fig. 1). Seasonal variation was evident for all age groups (Additional file 4: Figure S1; Additional file 5: Figure S2).

Among hospitalized adults with pneumonia, the proportion with >3 co-morbidities was highest among the age groups ≥65 years of age; approximately 50% of adults aged 65–74 and 75–84 had >3 co-morbidities. Having a co-morbidity approximately doubled the risk of ICU admission in all age-groups (Table 3). The risk of ICU admission did not change significantly with increasing number of comorbidities. Age was found to be an effect modifier, therefore; all analyses were stratified by age-group. On multivariable analysis, respiratory disease (excluding asthma) was an independent risk factor associated with ICU admission in all age-groups (Table 4). Malignancy was a risk factor for people aged <75 years, while male sex and immunosuppressive disorders were only risk factors for those aged ≥85 years. Neurological/musculoskeletal disorders and liver disease were risk factors for some age-groups. Asthma was a protective factor for ICU admission in people aged <75 years. The risk of ICU admission associated with diabetes was not independent of the risk associated with cardiovascular disease. However, after accounting for them in the multivariable model, both comorbidities were still risk factors for ICU admission in all age-groups, and for most age-groups in people who presented both comorbidities.

**Table 3:** Number of comorbidities associated with ICU admission in patients hospitalized with pneumonia —Vaccine Safety Data Link (VSD), 2006–2010

Age-groups and number of comorbidities	Number in comorbidity strata (% within age group)	No ICU admission (*n* = 96,879) No. (row %)	ICU Admission (*n* = 22,658) No. (row %)	Odds ratio (95% CI)
18–49 years (*n* = 13,999)		11,429 (81.6)	2570 (18.4)	
0 co-morbidities	3687 (26.3)	3243 (88.0)	444 (12.0)	REF
1 co-morbidity	5630 (40.2)	4474 (79.5)	1156 (20.5)	1.89 (1.68–2.12)
2 co-morbidities	2205 (15.8)	1758 (79.7)	447 (20.3)	1.86 (1.61–2.14)
≥ 3 co-morbidities	2477 (17.7)	1954 (78.9)	523 (21.1)	1.95 (1.70–2.24)
50–64 years (*n* = 24,893)		19,666 (79.0)	5227 (21.0)	
0 co-morbidities	2855 (11.5)	2517 (88.2)	338 (11.8)	REF
1 co-morbidity	8376 (33.6)	6574 (78.5)	1802 (21.5)	2.04 (1.80–2.31)
2 co-morbidities	5272 (2.21)	4099 (77.7)	1173 (22.3)	2.13 (1.87–2.43)
≥ 3 co-morbidities	8390 (33.7)	6476 (77.2)	1914 (22.8)	2.20 (1.94–2.49)
65–74 years (*n* = 24,724)		19,541 (79.0)	5183 (21.0)	
0 co-morbidities	1203 (4.9)	1076 (89.4)	127 (10.6)	REF
1 co-morbidity	5815 (23.5)	4639 (79.8)	1176 (20.2)	2.15 (1.77–2.61)
2 co-morbidities	5867 (23.7)	4628 (78.9)	1239 (21.1)	2.27 (1.87–2.75)
≥ 3 co-morbidities	11,839 (47.9)	9198 (77.7)	2641 (22.3)	2.43 (2.01–2.94)
75–84 years (*n* = 32,880)		26,870 (81.7)	6010 (18.3)	
0 co-morbidities	1228 (3.7)	1100 (89.6)	128 (10.4)	REF
1 co-morbidity	6765 (20.6)	5601 (82.8)	1164 (17.2)	1.79 (1.47–2.17)
2 co-morbidities	8363 (25.4)	6853 (81.9)	1510 (18.1)	1.89 (1.56–2.29)
≥ 3 co-morbidities	16,524 (50.3)	13,316 (80.6)	3208 (19.4)	2.07 (1.72–2.50)
≥85 years (*n* = 23,041)		19,373 (84.1)	3668 (15.9)	
0 co-morbidities	1089 (4.7)	997 (91.6)	92 (8.4)	REF
1 co-morbidity	5757 (25,0)	4865 (84.5)	892 (15.5)	1.99 (1.59–2.49)
2 co-morbidities	6253 (27.1)	5256 (84.1)	997 (15.9)	2.06 (1.64–2.57)
≥ 3 co-morbidities	9942 (43.1)	8255 (83.0)	1687 (17.0)	2.21 (1.78–2.76)

ICU = Intensive care unit

**Table 4:** Adjusted odds ratios for ICU admission for specific comorbidities in patients hospitalized with pneumonia, by age-group

High risk condition	Age 18–49 Adjusted OR (95% CI)	Age 50–64 Adjusted OR (95% CI)	Age 65–74 Adjusted OR (95% CI)	Age 75–84 Adjusted OR (95% CI)	Age ≥ 85 Adjusted OR (95% CI)
Male sex	---	---	---	---	1.11 (1.03–1.19)
Asthma	0.85 (0.76–0.95)	0.81 (0.75–0.88)	0.88 (0.81–0.96)	---	---
Respiratory diseases (excluding asthma)	1.33 (1.20–1.47)	1.12 (1.05–1.20)	1.10 (1.03–1.17)	1.14 (1.05–1.18)	1.09 (1.01–1.18)
Immunosuppressive disorders	---	---	---	---	1.27 (1.10–1.48)
Malignancy	1.21 (1.05–1.40)	1.16 (1.07–1.25)	1.15 (1.07–1.23)	---	---
Neurological/Musculosqueletal	1.42 (1.27–1.60)	1.10 (1.01–1.19)	---	0.92 (0.87–0.98)	---
Liver disease	---	1.16 (1.02–1.32)	---	---	---
Diabetes and cardiovascular disease					
Diabetes without cardiovascular disease	1.49 (1.32–1.68)	1.46 (1.34–1.59)	1.48 (1.33–1.64)	1.43 (1.28–1.59)	1.54 (1.31–1.81)
Cardiovascular disease without diabetes	1.49 (1.32–1.68)	1.40 (1.29–1.52)	1.39 (1.28–1.52)	1.24 (1.14–1.34)	1.31 (1.19–1.44)
Diabetes and cardiovascular disease	---	1.35 (1.24–1.48)	1.44 (1.32–1.57)	1.45 (1.33–1.57)	1.42 (1.26–1.60)

“---”= Not included in final model, ICU = Intensive care unit

Hemoglobinopathies and renal disease dropped out of the final model in every age-group and therefore are not shown

## Discussion

During 2006 through 2010, approximately 19% of hospitalizations with pneumonia had an ICU admission among adults enrolled in 6 managed care organizations. We found rates of 400 and 76 per 100,000 population/year for pneumonia hospitalizations and pneumonia hospitalizations with ICU admission, respectively. The highest burden of pneumonia hospitalizations and hospitalizations with ICU admissions was among persons aged ≥85 years; this group had rates 53 times higher for pneumonia and influenza hospitalizations, and 46 times higher for ICU admissions, compared to persons in the 18–49 age-group. Having a co-morbid condition approximately doubled the risk of an ICU admission associated with a pneumonia hospitalization.

Studies evaluating the incidence of severe pneumonia for adults are limited and the age-group specific rates for pneumonia hospitalization with ICU admission and assisted ventilation that we report are unique. Some studies have used ICD-9-CM codes to identify critical respiratory illness [7]; however, data on ICU admission are not reported in most administrative data sets. In addition, VSD data are unique in that they capture data from all enrolled adult members. Thus, unlike other databases, we were not limited to older adults with Medicare coverage or to datasets with all adults but with a subset of older adults with only certain types of Medicare insurance.

We found a similar proportion of pneumonia hospitalizations with ICU admission as reported by Jain et al. in a prospective study enrolling hospitalized patients with community acquired pneumonia (21% and 19%) [8], but higher than other studies reporting that approximately 10% of patients hospitalized for community acquired pneumonia are admitted to the ICU [9–12]. This difference might be related to our pneumonia case definition that included admissions with primary diagnoses of sepsis or respiratory failure with pneumonia as secondary diagnosis. In our data, 12% of hospitalized patients with a primary diagnosis of pneumonia required an ICU admission, compared to 36% of those with sepsis and 46% of those with respiratory failure as the primary diagnoses, with pneumonia as a secondary diagnosis. Our decision to use this expanded definition of pneumonia is supported by recent literature suggesting that coding practices have changed in recent years, and that this expanded definition is better for identifying patients with pneumonia, in comparison to only using a primary discharge diagnosis of pneumonia, which may miss more severe cases [5, 13, 14]. Our finding of a lower proportion of ICU admissions in people aged ≥85 years is in agreement with previously observed trends of a progressive decline in the proportion of pneumonia cases that are admitted to the ICU in older age-groups, though rates remain elevated compared with other age groups [15]. It is also consistent with an observed decrease in ICU utilization (all diagnoses) in older age-groups; more so in people aged ≥85 years [16, 17]. These trends may be related to increased hospitalization for pneumonia of lower severity with increasing age or less aggressive treatment among older individuals due to advance directives and living wills.

While several studies have reported rates for pneumonia hospitalizations, our rates may not be directly comparable to all published rates due to differences in age distributions and case definitions for pneumonia [5, 8, 15, 18, 19]. Our rates for pneumonia hospitalizations were similar to the adult age-group specific rates reported by Griffin et al. [20] during the 2007–2009 period. They used the National Inpatient Sample, a large all-payer nationwide inpatient care database, and defined pneumonia using ICD-9-CM codes (primary discharge diagnosis of pneumonia, or primary diagnosis of meningitis, septicemia or empyema, with a secondary diagnosis of pneumonia). Jain et al. reported a hospitalization rate for radiographically confirmed pneumonia of 248 per 100,000 in a prospective study enrolling hospitalized patients with community acquired pneumonia [8]; this rate is lower than our estimate (400 per 100,000) and may reflect the non-specific case definition for pneumonia that we used or issues related to defining radiologically confirmed pneumonia. Several studies have used administrative data to report rates of pneumonia hospitalizations [5, 15, 18, 19]. However, either the rates were higher and from earlier years [5], or the study used only first listed ICD-9-CM codes 480–486 for their definition of pneumonia [15, 18, 19]. Similar to our study, higher rates of pneumonia hospitalizations have been reported in older age-groups [15, 18] and older age is also associated with more serious outcomes [21, 22]. Our rates for pneumonia hospitalization for the ≥65 age-group were comparable with other reports [15, 18].

In our cohort, patients with any comorbidity were more likely to have ICU admission. The presence of comorbidities has been reported to be an important determinant of the need for ICU admission in patients with community acquired pneumonia [9].

Overall, 13% of patients with a pneumonia hospitalization and 42% of patients with an ICU admission associated with pneumonia received assisted ventilation. Our findings are generally consistent with other reports although we captured assisted ventilation (noninvasive and invasive mechanical ventilation) rather than focusing on invasive mechanical ventilation. In Jain et al., 6% of adults hospitalized with radiographically confirmed community acquired pneumonia required invasive mechanical ventilation [8]. A recent study assessing a small cohort of pneumonia patients admitted to the ICU, reported that 38.6% received mechanical ventilation [23]. In another study of patients with community acquired pneumonia in Spain, 44% of those admitted to the ICU received mechanical ventilation [24]. Similar to the trends that we observed for ICU admissions, the proportion of admissions receiving assisted ventilation decreased in older age-groups, while the rate increased with increasing age. The upward trend in rates associated with older age has been identified in previous publications focusing on mechanical ventilation (all causes) [25, 26]. The decreasing proportion of pneumonia hospitalizations that receive assisted ventilation among increasingly older adults is also similar to other reports [15].

There are several potential limitations to this study. We used discharge diagnosis codes to identify pneumonia hospitalizations, a standard but non-specific case definition for pneumonia. One consequence is our inability to differentiate between community-acquired and hospital-acquired pneumonia and the lack of radiology to confirm pneumonia. Although we present data from a large cohort collected from six sites throughout the U.S., most of the data are outcomes from two organizations in California (78% of pneumonia hospitalizations). This might limit the generalizability of our findings to the general U.S. population. In addition, we did not validate the capture of ICU admissions; this was the first attempt to capture ICU admissions in this system and each site developed new programs to query the electronic medical record for ICU admissions. In addition, our estimates of assisted ventilation used procedure codes to identify assisted ventilation, and were not verified for completeness or accuracy with direct chart reviews. We were also unable to differentiate between invasive and non-invasive assisted ventilation (i.e. persons intubated and mechanically ventilated versus persons receiving support from BIPAP or CPAP) which is a distinction that carries clinical relevance.

## Conclusion

In conclusion, we identified a significant burden of pneumonia hospitalizations requiring ICU admission during the study period. The highest rates for ICU admission were seen among older age-groups, and having underlying medical conditions was associated with ICU admissions. Obtaining ICU admission data from electronic medical records improved our ability to characterize pneumonia hospitalizations and our results could facilitate planning for resource utilization (i.e. use of hospital beds, ICU beds, and ventilators) during emergencies.

## Additional files

Additional file 1: Table S1. Current Procedural Terminology (CPT) and ICD-9-CM Procedure Codes for Assisted ventilation, Vaccine Safety Data Link (VSD), 2006–2010. (DOCX 30 kb)

Additional file 2: Table S2. Current Procedural Terminology (CPT) and ICD-9-CM Procedure Codes for comorbidities, Vaccine Safety Data Link (VSD), 2006–2010. (DOCX 32 kb)

Additional file 3: Table S3. Number and proportion of hospitalizations with primary diagnosis of pneumonia, primary sepsis diagnosis with secondary pneumonia, or primary respiratory failure diagnosis with secondary pneumonia —Vaccine Safety Data Link (VSD), 2006–2010. (DOCX 41 kb)

Additional file 4: Figure S1A. Monthly rates of pneumonia with an ICU admission by age-group—Vaccine Safety Data Link (VSD), 2006–2010. (DOCX 90 kb)

Additional file 5: Figure S1B. Monthly rates of pneumonia with an ICU admission for age-groups 18–49 and 50–64. —Vaccine Safety Data Link (VSD), 2006–2010. (DOCX 76 kb)

## Abbreviations

CDC: Centers for Disease Control and Prevention
CI: confidence intervals
CPT: current procedure terminology
ICD-9-CM: Classification of Diseases, Ninth Revision, Clinical Modification
ICU: intensive care unit
IQR: interquartile range
VSD: vaccine safety datalink
