# Supplementary material for: Rates and risk factors associated with hospitalization for pneumonia with ICU admission among adults
Source: BMC Pulm Med. 2017 Dec 16;17:208. doi: 10.1186/s12890-017-0552-x (PMC5732529; doi:10.1186/s12890-017-0552-x)
Supplement: Supplementary file 1 — Current Procedural Terminology (CPT) and ICD-9-CM Procedure Codes for Assisted ventilation, Vaccine Safety Data Link (VSD), 2006–2010. (DOCX 30 kb) [file 12890_2017_552_MOESM1_ESM.docx]

Additional file 1: Table S1. Current Procedural Terminology (CPT) and ICD-9-CM Procedure Codes for Assisted ventilation, Vaccine Safety Data Link (VSD), 2006–2010

| **Current Procedural Terminology and ICD-9-CM Procedure Codes** | **Procedures** |
| --- | --- |
| **Current Procedural Terminology Codes** | |
| 31500 | Intubation, endotracheal, emergency procedure |
| 36822 | ECMO |
| 94002 | Mechanical ventilation, hospital inpatient/observation, initial day |
| 94003 | Mechanical ventilation, hospital inpatient /observation, each subsequent day |
| 94004 | Mechanical ventilation, initiation and management, per day |
| 94656 | Ventilation assist & management, first day |
| 94657 | Ventilation assist & management, subsequent days |
| 94660 | CPAP (continuous positive airway pressure) initiation and management |
| 94662 | CNP (continuous negative pressure) initiation and management |
| **ICD-9-CM Procedure Codes** | |
| 39.65 | ECMO |
| 93.90 | non-invasive mechanical ventilation |
| 93.91 | Intermittent positive pressure breathing |
| 96.01 | insertion of nasopharyngeal airway |
| 96.02 | insertion of oropharyngeal airway |
| 96.04 | Insertion of endotracheal tube |
| 96.05 | Insertion of respiratory tract |
| 96.7 | Other continuous mechanical ventilation |
| 96.70 | Continuous mechanical ventilation of unspecified duration Mechanical ventilation not otherwise specified |
| 96.71 | Continuous mechanical ventilation for less than 96 consecutive hours |
| 96.72 | Continuous mechanical ventilation for 96 consecutive hours or more |
