# Supplementary material for: Rates and risk factors associated with hospitalization for pneumonia with ICU admission among adults
Source: BMC Pulm Med. 2017 Dec 16;17:208. doi: 10.1186/s12890-017-0552-x (PMC5732529; doi:10.1186/s12890-017-0552-x)
Supplement: Supplementary file 2 — Current Procedural Terminology (CPT) and ICD-9-CM Procedure Codes for comorbidities, Vaccine Safety Data Link (VSD), 2006–2010. (DOCX 32 kb) [file 12890_2017_552_MOESM2_ESM.docx]

Additional file 2: Table S2. Current Procedural Terminology (CPT) and ICD-9-CM Procedure Codes for comorbidities, Vaccine Safety Data Link (VSD), 2006–2010

| **Comorbidities** | **ICD-9-CM codes** |
| --- | --- |
| Cardiovascular | 093, 391, 392, 393, 394, 395, 396, 397, 398, 402, 404, 410, 411, 412, 413, 414, 416, 417, 421, 423, 424, 425, 428, 429, 440, 446, 745, 746, 747, 427.1, 427.2, 427.3, 427.4, 427.5, 427.8, V42.1, V45.0, V43.3, 112.81, V45.81, V45.82, 442, 443, 444, 447, 446.1, 438, 430, 431, 432, 433, 434, 435, 436, 437 |
| Respiratory | 491, 492, 493, 494, 496, 273.4, 011, 012, 135, 495, 500, 501, 502, 503, 504, 505, 506, 508, 510, 513, 514, 515, 516, 517, 769, 770, 031.0, 277.0, 507.0, 507.1, 518.0, 518.1, 518.2, 518.3, 518.8, 519.9, 519.0, 748.4, 748.5, 748.6, 759.3, 770.2, 770.7, V42.6, 714.81 |
| Renal | 585, V56, V45.1, 285.21, 403, 581, 582, 583, 586, 587, 588.0, 588.1, 590.0, 593.8 |
| Hepatic | 571, 572.1, 572.2, 572.3, 572.4, 572.5, 572.6, 572.7, 572.8 |
| Immunosuppressive | 042, 043, 044, V08, V42.0, V42.2, V42.4, V42.7, V42.8, V42.9, V42.1, V42.6, 996.8, 279, 446, 710, 714, 555, 556, 759, 079.5, 136.3, 288.0, 288.1, 288.2, 710.0, 710.2, 710.4, 289.5, 298.4, 298.5, 963.1, 289.89 |
| Malignancy | 140, 141, 142, 143, 144, 145, 146, 147, 148, 149, 150, 151, 152, 153, 154, 155, 156, 157, 158, 159, 160, 161, 162, 163, 164, 165, 166, 167, 168, 169, 170, 171, 172, 174, 175, 176, 177, 178, 179, 180, 181, 182, 183, 184, 185, 186, 187, 188, 189, 190, 191, 192, 193, 194, 195, 196, 197, 198, 199, 200, 201, 202, 203, 204, 205, 206, 207, 208, 235, 236, 237, 238, V58.0, V58.1, 960.7 |
| Neurological/musculoskeletal | 271, 315, 317, 318, 319, 290, 330, 331, 334, 335, 340, 341, 343, 438, 345, 348, 438, 356, 741, 742, 294.1, 318.1, 318.2, 333.0, 333.4, 333.5, 333.6, 333.7, 333.8, 333.9, 344.0, 358.0, 358.1, 359.1, 359.2, 756.4, 780.3, 277.2, 277.5, 783.42 |
| Metabolic | 250, 251, 648.0, 357.2, 362.0, 362.11, 366.41, 278, 255, 270, 271, 277.2, 277.3, 277.5, 277.8, 272.1, 272.2, 272.3, 272.5, 272.6, 272.7, 272.8, 272.9, 277.1, 272.6, 277.9 |
| Hemoglobinopathies | 282, 283, 284 |
| Pregnancy | 640, 641, 642, 643, 644, 645, 646, 647, 648, 765, 767, 768, 758, 759, V22, V23, V24, V27, V28, 771.0, 771.1, 771.2, 724.2, 277.8 |
