# Supplementary material for: Rates and risk factors associated with hospitalization for pneumonia with ICU admission among adults
Source: BMC Pulm Med. 2017 Dec 16;17:208. doi: 10.1186/s12890-017-0552-x (PMC5732529; doi:10.1186/s12890-017-0552-x)
Supplement: Supplementary file 3 — Number and proportion of hospitalizations with primary diagnosis of pneumonia, primary sepsis diagnosis with secondary pneumonia, or primary respiratory failure diagnosis with secondary pneumonia —Vaccine Safety Data Link (VSD), 2006–2010. (DOCX 41 kb) [file 12890_2017_552_MOESM3_ESM.docx]

Additional file 3: Table S3. Number and proportion of hospitalizations with primary diagnosis of pneumonia, primary sepsis diagnosis with secondary pneumonia, or primary respiratory failure diagnosis with secondary pneumonia —Vaccine Safety Data Link (VSD), 2006–2010

|  | **Hospitalizations** | **ICU admissions** | **Assisted ventilation** |
| --- | --- | --- | --- |
| **Demographic variable** | **No. (% of total)** | **No. (% of hosp.)** | **No. (% of hosp.)** |
| **Primary pneumonia hospitalization** |  |  |  |
| **Overall** | 87457 (100) | 10191 (11.7) | 5780 (6.6) |
| **Age-groups (years)** |  |  |  |
| 18 – 49 | 10726 (12.3) | 1212 (11.3) | 706 (6.5) |
| 50 – 64 | 18167 (20.8) | 2350 (12.9) | 1472 (8.1) |
| 65 – 74 | 17489 (20.0) | 2190 (12.5) | 1312 (7.5) |
| 75 – 84 | 24105 (27.6) | 2742 (11.4) | 1505 (6.2) |
| ≥85 | 16970 (19.4) | 1697 (10.0) | 785 (4.6) |
| **Sex** |  |  |  |
| Male | 43017 (49.2) | 5202 (12.1) | 3047 (7.1) |
| Female | 44435 (50.8) | 4989 (11.2) | 2732 (6.2) |
| **Primary sepsis and secondary pneumonia hospitalization** |  |  |  |
| **Overall** | 23389 (100) | 8451 (36.1) | 5250 (22.4) |
| **Age-groups (years)** |  |  |  |
| 18 – 49 | 2432 (10.4) | 951 (39.1) | 593 (24.4) |
| 50 – 64 | 4659 (19.9) | 1903 (40.8) | 1281 (27.5) |
| 65 – 74 | 4946 (21.1) | 1903 (38.5) | 1289 (26.1) |
| 75 – 84 | 6451 (27.6) | 2200 (34.1) | 1325 (20.5) |
| ≥85 | 4898 (20.9) | 1494 (30.5) | 762 (15.6) |
| **Sex*** |  |  |  |
| Male | 11788 (50.4) | 4353 (36.9) | 2746 (23.3) |
| Female | 11601 (49.6) | 4098 (35.3) | 2504 (21.6) |
| **Primary respiratory failure and secondary pneumonia hospitalization** |  |  |  |
| **Overall** | 8691 (100) | 4016 (46.2) | 4534 (52.2) |
| **Age-groups (years)** |  |  |  |
| 18 – 49 | 841 (9.7) | 407 (48.4) | 473 (56.2) |
| 50 – 64 | 2067 (23.8) | 974 (47.1) | 1109 (53.7) |
| 65 – 74 | 2289 (26.3) | 1090 (47.6) | 1242 (52.3) |
| 75 – 84 | 2321 (26.7) | 1068 (46.0) | 1176 (50.7) |
| ≥85 | 1173 (13.5) | 477 (40.7) | 534 (45.5) |
| **Sex*** |  |  |  |
| Male | 4178 (48.1) | 1953 (46.7) | 2225 (53.2) |
| Female | 4512 (51.9) | 2062 (45.7) | 2308 (51.2) |
| * Sex was missing for 6 persons |  |  |  |
