# Supplementary material for: Rates and risk factors associated with hospitalization for pneumonia with ICU admission among adults
Source: BMC Pulm Med. 2017 Dec 16;17:208. doi: 10.1186/s12890-017-0552-x (PMC5732529; doi:10.1186/s12890-017-0552-x)
Supplement: Supplementary file 4 — Monthly rates of pneumonia with an ICU admission by age-group—Vaccine Safety Data Link (VSD), 2006–2010. (DOCX 90 kb) [file 12890_2017_552_MOESM4_ESM.docx]

Additional file 4: Figure S1A. Monthly rates of pneumonia with an ICU admission by age-group—Vaccine Safety Data Link (VSD), 2006–2010


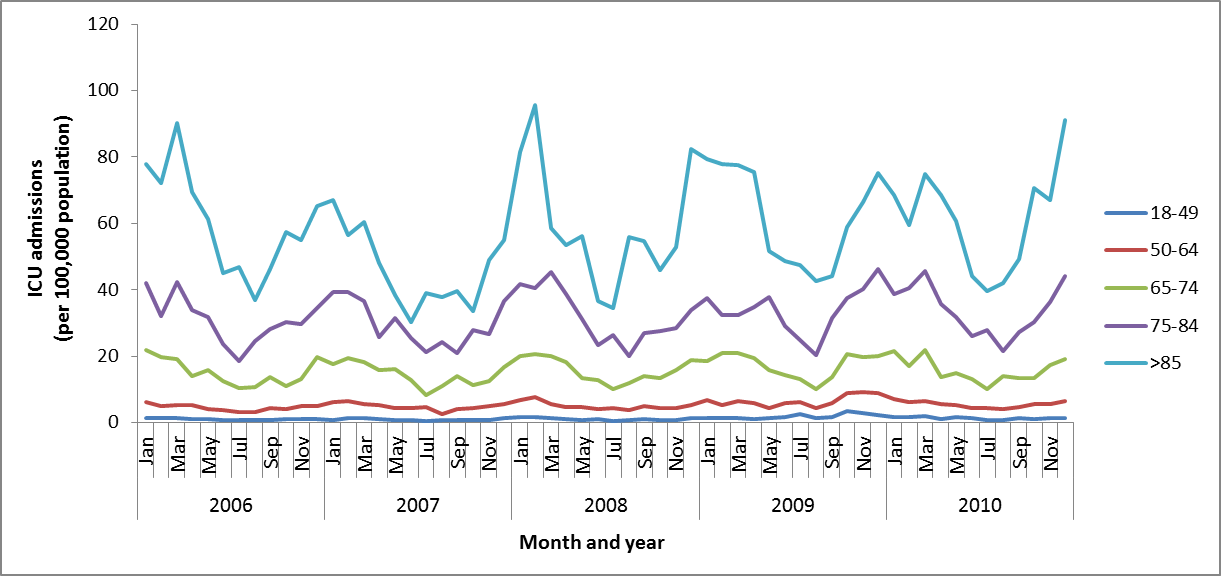


**Age-group**

**(years)**

ICU = Intensive care unit
