# Supplementary material for: Rates and risk factors associated with hospitalization for pneumonia with ICU admission among adults
Source: BMC Pulm Med. 2017 Dec 16;17:208. doi: 10.1186/s12890-017-0552-x (PMC5732529; doi:10.1186/s12890-017-0552-x)

Additional file 5: Figure S1B. Monthly rates of pneumonia with an ICU admission for age-groups 18 – 49 and 50 – 64. —Vaccine Safety Data Link (VSD), 2006–2010


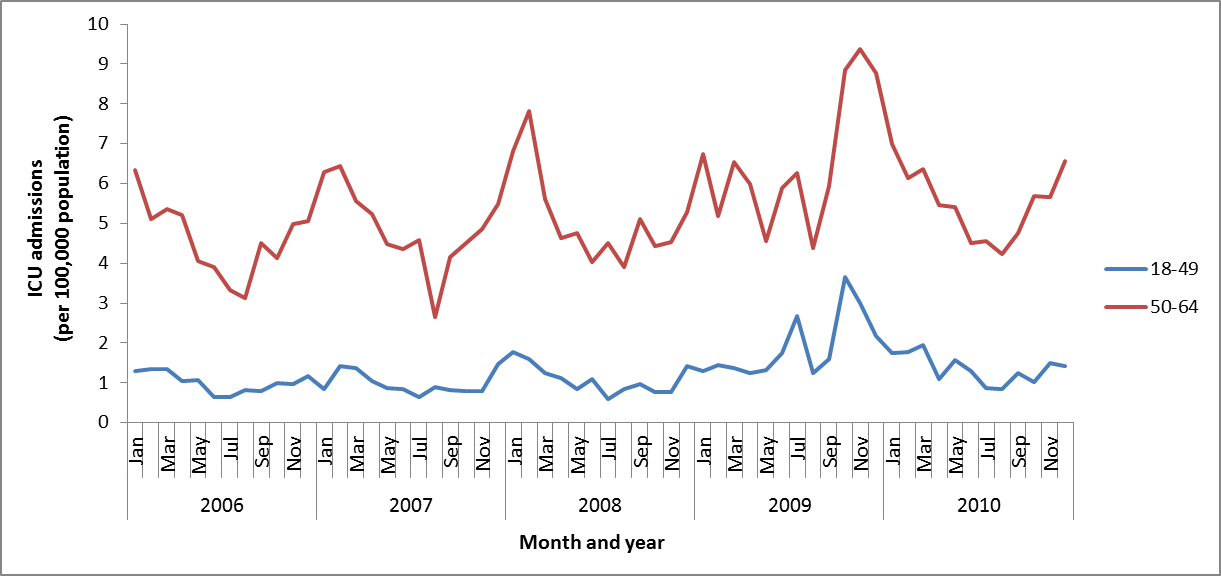

Supplement: Supplementary file 5 — Monthly rates of pneumonia with an ICU admission for age-groups 18–49 and 50–64. —Vaccine Safety Data Link (VSD), 2006–2010. (DOCX 76 kb) [file 12890_2017_552_MOESM5_ESM.docx]
